# Experience with Support at Workplaces for People with Young Onset Dementia: A Qualitative Evaluation of Being Open about Dementia

**DOI:** 10.3390/ijerph20136235

**Published:** 2023-06-27

**Authors:** Shizuko Omote, Satomi Ikeuchi, Rie Okamoto, Yutaro Takahashi, Yoshiko Koyama

**Affiliations:** 1 Faculty of Health Science, Kanazawa University, Kodatsuno 5-11-80, Kanazawa 920-0942, Japan; sikeuchi@staff.kanazawa-u.ac.jp (S.I.); nrie@staff.kanazawa-u.ac.jp (R.O.); y-takahashi@staff.kanazawa-u.ac.jp (Y.T.); 2 Kinjo University, Kasama-machi 1200, Hakusan 924-8511, Japan; ykoyama@wing.ocn.ne.jp; 3 Ishikawa Occupational Health Support Center, Sainen 1-1-3, Kanazawa 920-0024, Japan

**Keywords:** young-onset dementia, employment, support, qualitative research, Japan

## Abstract

Young-onset dementia (YOD) occurs at <65 years of age. Individuals with YOD experience social and psychological disturbances, including a loss of employment. This economic toll affects them, their families, and their caregivers. Employers have an increasing role in supporting affected employees in remaining employed, an important component of the “fight for their dignity”. This study aims to clarify the workplace support experiences of employees with YOD. To assess the experiences of employers with at least one affected employee, we interviewed personnel from eight facilities for qualitative analysis. We identified 5 unique categories and 14 subcategories encompassing the following aspects: confusion at the workplace stemming from the uncertainty of the disease, sensitivity when recommending consultation and diagnosis, creating a system that considers safety and security, building consensus among employers, supporting employees with YOD and their families, and assisting the individual with YOD with activities of daily living. Employers should be open to addressing dementia-related issues in the workplace, including obtaining information from employees’ physicians so that appropriate support can be provided. Appropriate support can include work accommodations, needs-based support, and meetings with families to build consensus for continued employment. This information can facilitate the creation of new training materials for employers.

## 1. Introduction

Young-onset dementia (YOD) is defined as dementia occurring in patients aged <65 years old [1]. The causes of dementia include Alzheimer’s disease, vascular dementia, frontotemporal dementia, and secondary dementia, including cases resulting from infection, autoimmune diseases, or substance abuse. A meta-analysis conducted in 2002 showed that the age-standardized rate was 11/100,000 for individuals between the ages of 30 and 64 years, with an estimated 370,000 new cases globally [2].

In Japan, the standardized prevalence rates by area range from 25/100,000 to 64/100,000, with the highest rate in Ehime Prefecture [3]. This is comparable to rates in the United Kingdom, Australia, and Norway [4,5,6].

The Japanese Health Policy NOW published a policy paper on treating individuals with dementia in Japanese society [7]. The goal of this document is to “raise awareness and promote the understanding of dementia”. The aim is to create dementia-friendly communities by maintaining familiar environments for as long as possible. To support individuals with YOD, the plan calls for counseling services for them and their families, caretakers, and employers in each prefecture.

A meta-analysis [8] found several negative outcomes for individuals, their families, and their caretakers. A major impact of YOD is the loss of social interaction, leading to a diminished quality of life [9,10,11,12,13].

A significant challenge for individuals with YOD is the “fight for their dignity” [14]. Johannessen and Möller [14] used the term to describe facing “challenges in everyday life”, including both social and psychological challenges. Economic losses, including employment loss, are among the most significant changes. There is a significant financial burden for individuals with YOD and their caregivers [8,9,12]. An interview-based study in Ireland interviewed ten individuals with YOD and their family members [12] and found that four of the five participants who were employed at the time of their diagnosis were compelled to leave their jobs without compensation. Caregivers reported a significant increase in stress levels: for one person, stress led to early retirement, and for another, a change in work duties [12]. Evans et al., (2019) found that the onset of symptoms leads to problems at work, including increased errors, poor time management, and disorganization [9].

In Japan, the Labor Contracts Act [15] and the Industrial Safety and Health Act [16] stipulate that employers have “an obligation to ensure workers’ health and safety” and “must also work to ensure the safety and health of workers in the workplace through the creation of a comfortable work environment and the improvement of working conditions”. Occupational physicians, whose duties include managing employees’ health, urge employees to undergo examinations at a medical institution if they deem them necessary based on past health checkups. With employee consent, occupational physicians can also obtain information from their physicians at medical institutions when job performance is affected, and there is a requirement in the workplace to help workers balance treatment and work.

There are many benefits to providing significant support to individuals with YOD and their caregivers [7]. In 2021, a guide on balancing treatment and work for individuals with YOD was published [17]. However, it is often difficult for employees with YOD to continue working after being diagnosed because of the early effects of the disease. Although there are examples of companies that support the continued employment of people with early-onset dementia, the majority still lack processes for integrating these employees into the workforce [18].

Supportive measures, such as continuing routine activities and maintaining community engagement and interaction, can help manage the stress levels of patients and their caretakers. Overall, research on how employers can support individuals with YOD can improve the quality of life for affected individuals. This study aims to clarify the support experiences in workplaces where employees with YOD work.

## 2. Methods

### 2.1. Participants

We identified 10 individuals with YOD from eight workplaces in the Tohoku, Hokuriku, and Kyushu/Okinawa areas of Japan and interviewed their supervisors or occupational health staff with their employers’ consent (*n* = 10). In a previous study, Omote et al. asked human resource managers of the subject workplaces if they were willing to participate in an interview regarding their support for individuals with YOD [19]. Two of the seven facilities identified by Omote had employees willing to participate in interviews [19]. It was difficult to find workplaces with experience in providing employment support to employees with YOD. Therefore, we asked our research team members and colleagues to search for workplaces with employees with YOD. In addition, the researcher asked a supporter of people with YOD, who was an acquaintance of the researcher, to refer her to an employer.

Interviews were conducted with personnel or occupational health staff with the employer’s consent. A qualitative and descriptive approach was adopted to comprehensively describe this phenomenon [20,21]. We used a semi-structured interview method with the identified workplace participants. We asked the participants about workplace support for employees who were diagnosed with early-onset dementia.

### 2.2. Ethics

The study was approved by the Kanazawa University Medical Ethics Review Board (approval no. 749-1, dated 24 January 2017) and was conducted in accordance with the principles of the Declaration of Helsinki.

A request form describing the study’s purpose, methods, and ethical considerations was sent to the individuals with YOD and their supervisors at the workplace.

Prior to the interviews, the purpose, methods, and ethical considerations of the study were explained, and the participants provided written informed consent to publish their anonymized information in the manuscript. Participants were assured that their participation in the study would be voluntary, that they would not be disadvantaged by refusing to participate, and that their privacy and personal information would be protected.

### 2.3. Data Analysis

Qualitative descriptive analysis was used to codify the interviews [20]. A verbatim transcription was performed. The data were read multiple times to determine participants’ experiences with supportive measures. The first and second authors read the transcripts and analyzed the data. Subcategories were derived based on similarities and differences in the semantic content. Seven categories were created based on the subcategories of workplace experiences. The final results of the category creation process were discussed among the researchers.

## 3. Results

The eight workplaces from where participants were recruited included establishments engaged in manufacturing, healthcare and welfare, transportation, education, and temporary staffing (Table 1). Each workplace had at least one individual with YOD. Interviews were conducted with ten supervisors or occupational health staff. We identified 5 categories and 14 subcategories from 34 codes related to support experiences in the workplaces where employees with YOD were working. Table 2 presents the interview results. The main results are shown by category. Italics represent the participants’ own words.

### 3.1. Confusion in the Workplace Stemming from the Uncertainty of the Disease

We identified a subcategory in which the employer experienced uncertainty regarding interacting with an employee with YOD. Employers reported observing changes in the appearance of their employees but did not suspect dementia. The differences identified included employees increasingly forgetting and repeating tasks in recent years or a change in the task completion rate. Employers were uncertain about how to communicate their observations, such as bringing forgetfulness to the attention of affected employees. Employer contacts were reported to have sought guidance on how to engage with co-workers with YOD.

Co-workers often saw changes in the employees’ performance and accuracy but did not know the full situation. This lack of information led to undesirable speculation about the employee’s health status. For example:

“*His writing has become poor. He can’t do the work he could always do before. Small tasks take time. He talks less, and only in old stories or things he is good at he talks well. He also can’t recall the names of members at the workplace. He can’t remember new things, so everyone was worried that he might have dementia, so everyone came to discuss with me*.”

(Case D)

And

“*He told me something like that, if he only says a little bit, people will surely say various things about what is or isn’t there, and because of that, he didn’t want to say it to too many people*.”

(Case B)

### 3.2. Sensitivity when Recommending Consultation and Diagnosis

Employers also reported heightened sensitivity when recommending consultations with medical professionals. Affected employees may not be receptive to employers highlighting new issues related to work products. Employers indicated that management could look for opportunities to recommend medical examinations or offer appropriate personnel (e.g., company nurses) to accompany employees to the hospital or clinic or provide support after their visit. When recommending medical visits, the employer can use errors as an opportunity to open that conversation (e.g., “*When I said, ‘everyone is so worried, so let’s go to the hospital’, and then he said, ‘I understand*’” (Case D)). A diagnosis is an opportunity to think together about work options.

“*The person herself also apparently felt worried about something for a long time, so she said, ‘then I would go to see the doctor’. Then the person herself said, ‘if I go, I want to go here,’ and when I said, ‘do you want to go with your husband?’ she said, ‘I want you to come together with me’ so I went together with her for examination*.”

(Case E)

### 3.3. Creating a System That Considers Safety and Security

Employers also reported that understanding the diagnosis in detail allowed them to work with employees on alternative duties when work cannot be completed as instructed. Managers can also evaluate workspaces and environments for potential hazards. Regular meetings with occupational doctors can provide support and information regarding changes in work duties.

“*The way we are currently working in the field is that we have organized that we are always working with someone. The partner who pairs with has already become the person in charge, and I have already told everyone about the person in charge. In such a situation, we are proceeding in the form of ‘I want you to work together. While covering each other’*.”

(Case I)

The workplaces supported employees whenever possible. Staff could provide feedback for management, including shifts in responsibilities to other employees.

“*As for employment, of course, we employed him as a regular employee at first, but the person himself says that it ultimately is overwhelming and tiring, so partway through, we reduced the hours a bit and made it a bit like a contract worker*.”

(Case F)

The employers had processes that ensured that employees continued working at their capacity by discussing work decisions and respecting their pride in their work and experience. Another subcategory was used to avoid assigning new tasks that needed to be remembered.

“*It’s also tentatively the corporate culture to do while always communicating with the person himself/herself. In such circumstances, since T himself/herself and the person himself were actually diagnosed with dementia, it is not bad implications for the person himself, but of course, while having him understand the risks, the company wanted him to work a little bit while taking a back-up system in this way as a company, and we are proceeding after obtaining approval*.”

(Case I)

“*We really want the person herself to work comfortably after all, and it is unthinkable at all to make what was her pride for 5, 10 years, what she had for herself like nothing*.”

(Case A)

### 3.4. Building Consensus between the Workplace, Employees with YOD, and Their Families

Employers should consider supporting meeting opportunities that allow family members to arrive at the workplace with their consent. These meetings can provide insights into what the family knows about an employee’s condition and how they accept it.

“*What we want to know as the business facility side may be different from what the person himself or his family wants to know, and hearing what the person himself and his family may have heard, there may be cases that it may not match what the business facility wants to know*.”

(Case F)

Meetings can provide opportunities to discuss potential departmental transfers due to employees’ declining performance. By including the family in discussions, employees can explore the best options, including whether there should be a change in work location.

“*After we had his family come, he was in the workplace there for one or two months, but after that, it was decided that we would have him come to the general affairs department, do the work that he could do at the general affairs department*.”

(Case B)

### 3.5. Supporting the Person with Activities of Daily Living

Supporting employees with YOD includes discussions on leave periods and illness or accident benefits. An employer reported that they had introduced feasible workplace systems, including suggesting an application for a disability pension. Employers also assisted employees in finding safe commuting methods.

“*I was asked, “what kind of system does the company have?”, and I provided specifics, like, there’s this long leave period and so on, and this much disability allowance, and then the person himself went home, discussed with his family, and went on leave*.”

(Case B)

“*After the illness was discovered, we continued to employ him as is until the handbook was issued and pension was confirmed, and we have decided that, after pension was fixed, we would change his employment status according to the person himself’s [sic] status*.”

(Case F)

## 4. Discussion

Individuals in the early stages of YOD may face workplace challenges before a formal evaluation and/or diagnosis. The early signs of YOD are often nonspecific and can be easily overlooked or misinterpreted. Employers can interpret employees’ increased forgetfulness and disorganization as poor work performance, leading to a reduction in duties, exclusion from workplace functions, and potential dismissal [9,22]. In situations where the employee’s diagnosis is unknown, co-workers in the department may feel uncertain about how to interact with the affected individual. However, if other employees report noticeable changes in the performance of an employee with YOD, the employer may arrange a meeting with an occupational physician and suggest an examination. This course of action is supported by the Employment Act and the Industrial Safety and Health Act of Japan. Once a diagnosis is confirmed, the employer will engage in discussions with the employee, aiming to provide support in achieving a balance between treatment and work responsibilities. In the INSPIRED Study, Draper et al., (2016) found that the median time from symptom onset to the first consultation was 2.3 years and 4.7 years to a final diagnosis [23]. Employers can reduce the time to diagnosis by providing information about cognitive changes to family members [24].

### 4.1. Being Open about Dementia

A diagnosis of YOD can create multiple sources of anxiety for patients and their families. Disclosing a YOD diagnosis at work can affect an individual’s continued employment, perhaps earlier than the deficits would [9,12,22,23]. It is important for the employer to receive the diagnosis and information from the physician so that appropriate support can be provided. In Japan, the guidelines for balancing medical treatment and work [17] present important points regarding the provision of information from medical institutions to support employees. As indicated in Section 3, it is crucial for employers to provide support to employees diagnosed with YOD by having their health staff accompany them to clinic visits and gathering information from their physicians regarding job performance and necessary considerations in the workplace.

However, anxiety about disclosing a diagnosis can be compounded by employers’ lack of knowledge about the management of employees with YOD [9,25,26]. Affected employees may be concerned about others’ reactions, including bullying [27,28]. Processes to support employees with YOD could include employers setting up meetings to build a consensus between the workplace, employees, and families with the employee’s consent. Employers can use this opportunity to confirm the family’s understanding and acceptance of an employee’s condition. An alternative would be to consider transferring employees to another department or work location.

### 4.2. Workplace Accommodations

In 2019, the Japanese government published a report on the Convention on the Rights of Persons with Disabilities [29]. In this document, the government defined the purpose of the Convention as follows.

“*The purpose of the present Convention is to promote, protect, and ensure the full and equal enjoyment of all human rights and fundamental freedoms by all persons with disabilities and to promote respect for their inherent dignity. Persons with disabilities include those who have long-term physical, mental, intellectual, or sensory impairments which in interaction with various barriers may hinder their full and effective participation in society on an equal basis with others*.”

This Convention calls for legislation prohibiting discrimination based on disability with regard to all matters concerning all forms of employment, including conditions of recruitment, hiring, and employment, the continuance of employment, career advancement, and safe and healthy working conditions. Companies should respect these principles regarding YOD.

Employers must create safe and secure workspaces for employees with early dementia [7,25,30]. Respondents suggested that meeting this requirement could include observing employees to reduce or avoid risks. The assistance of a co-worker or consultation with an occupational physician can be used to ensure that safety measures for all employees are optimized [31].

The workplaces in this study were small- and mid-sized companies, indicating that they would need additional support staff, including medical services and human resources, to support the continued employment of employees diagnosed with YOD [32]. A small number of employees may make it difficult to provide individualized support. Ideally, companies should have a plan to consult someone when employees will need additional support, including information regarding the employment of young people with dementia. Ideally, this plan should identify a place for consultation and the organizations involved.

This study has some limitations. First, we did not investigate the age, sex, or underlying disease of the patients with YOD; therefore, the results did not consider symptoms and individual characteristics. Second, the results of the present study cannot be generalized to other parts of the world. Our results reflect the experiences of employers in Japan. The modes of support provided by employers may differ across countries. For example, an employer attending medical appointments or inviting family members to the workplace is considered unusual in the United Kingdom. The normal practice of such aspects varies among cultures and could be influenced by the personal choices of a person with YOD. Additionally, contract-specific variability in employee rights may exist among countries, and different countries may have different levels of employee protection. Moreover, someone who is self-employed or has a short-term contract may receive very little support from the employer. Third, we did not consider other conditions such as behavioral variant frontotemporal dementia, which can cause behavioral changes in affected individuals, leading to severe workplace difficulties.

## 5. Conclusions

This study identified workplace support experiences for employees with YOD. In these workplaces, management adjusted work content and systems to allow employees to work easily. Employers’ support for medical examinations is also important because information about the diagnosis of employees with YOD enables their co-workers to deal with the affected employees and their families. Although there are challenges, acknowledging that an employee has YOD can reassure co-workers through open communication. Employers should support all workplace employees using both internal and external systems. Future research should create training content for the workplaces on YOD and evaluate the content and effectiveness of the training courses.

## Figures and Tables

**Table 1:** List of research participants (case providers).

Participant Number (*n* = 10)	Industry	Research Participant	Case			Number of Employees in Industry
			Age	Sex	Diagnosis Present/Absent	
A	Health and sanitation	Director of facility, in charge of personnel	60s	Female	Present	124
B	Manufacturing	Industrial nurse	60s	Male	Present	520
C	Manufacturing	Industrial nurse	50s	Female	Present	520
D	Transport and transportation	Industrial nurse	60s	Male	Present	100
E	Education and research	Director of facility	60s	Female	Present	30
F	Health and sanitation	Director of facility	50s	Male	Present	53
G	Health and sanitation	Department chief	50s	Male	Present	69
H	Temporary staffing	Department chief	50s	Male	Present	200
I	Temporary staffing	Department chief	60s	Male	Present	40
J	Sales	Department chief	40s	Male	Present	20

Case: Employee diagnosed with YOD.

**Table 2:** Experience of employees with young-onset dementia regarding support in workplaces.

Category	Subcategory	Code
Confusion in the workplace stemming from the uncertainty of the disease	Uncertainty of how to relate to employee with YOD	Paused to think when the progress of work is different from before, but work is done
		Unsure of whether to tell employee of forgetfulness
	If co-workers are only told of symptoms, they can only surmise what the disease is	Colleagues guess illness due to changes in the employee’s behavior
		Employee does not want incorrect information about them spread
		Co-workers can see that the employee is having difficulties at work
Sensitivity when recommending consultation and diagnosis	Look for opportunities to recommend medical examinations	Use examples of work mistakes as an opportunity to recommend medical consultation
		The person himself/herself hesitates to undergo a medical examination
		Recommend medical examination since the earlier, the better if treatment is needed
	Go with the employee to visit the hospital and give support after the visit	If an employee visits the hospital alone, the workplace may not understand the diagnosis and treatment well
		Nurse accompanies the employee to their medical examination at his/her request
	Diagnosis is an opportunity to think together about work options	Employer understands that the employee cannot work as usual after seeing medical certificate
		After the diagnosis, the employee’s family is convinced that the person himself/herself cannot work as usual
Creating a system that considers safety and security	Avoid risk by watching attentively	Enlist a co-worker who can take responsibility and assist
		Change work when it becomes difficult to do the work as instructed
		Judge work by looking at whether there are any hazards
		Manager regularly meets with the occupational doctor to confirm the employee’s condition
	Workplaces support employees wherever possible	Support employees until they decide that they can no longer work
		At all times, the staff reports on the status to the manager
		Management wants the employee to continue working but thinks it is a burden on other employees
	With the employee’s consent, disclose dementia to colleagues	Decide on the scope of public disclosure of illness with the consent of the person himself/herself
		Wait until employee is ready to disclose dementia to co-workers
	Consider a work setting where the person with young-onset dementia can make the most of his or her strengths	Confirm with the employee when planning
		Assign a job that the person is good at
		The company respects employee’s pride in their work and experience
	Avoid new tasks that need to be remembered	Have employees take charge of the same work as before
		Request work on a fixed schedule
Building consensus among workplace, person with young-onset dementia, and their family	Schedule a meeting	With the employee’s consent, family members are invited to come to the workplace
		Confirm extent of family’s understanding of employee’s condition
		Confirm how family is accepting illness
	Consider department transfer	Difficult to convey transfer due to declining ability
		Change work location after meeting with family
Supporting continued living in the future	Recommendation for an internal system that can be used until retirement	Explain leave period and accident and sickness benefits
	Suggesting the pension applications that will be required after retirement	Propose application for disability pension
		Connect with community social workers

## Data Availability

No new data were created or analyzed in this study. Data sharing is not applicable to this article.
